# Patient Characteristics Related to Blood Loss in High Tibial Osteotomy in Novel Multiple Linear Regression Analysis

**DOI:** 10.1155/2020/8965925

**Published:** 2020-05-01

**Authors:** Jia-Wang Zhu, De-Sheng Chen, Tong-Fu Wang, Yang Xie

**Affiliations:** Department of Tianjin Hospital, Tianjin, China

## Abstract

The purpose of the study was to identify patient characteristics related to blood loss following high tibial osteotomy (HTO). We evaluated 48 patients undergoing HTO from August 2018 to August 2019. The data of 48 patients were collected, including gender, age, height, weight, body mass index (BMI), smoking, alcohol consumption, hypertension, diabetes, history of aspirin, and pre-postoperative hematocrit (Hct). Multiple linear regression analysis was used to analyze the risk factors related to blood loss in HTO. The mean age of patients was 56.6 ± 10.2 years, including 22 males and 26 females. The mean BMI was 28.5 ± 4.2 kg/m^2^, and the mean blood loss volume was 383.3 ± 181.3 mL, 13 patients with smoking (27.1%), 15 patients with alcohol consumption (31.3%), 23 patients with hypertension (47.9%), 10 patients with diabetes mellitus (20.8%), and 12 patients with history of aspirin (25.0%). Multiple linear regression model suggested alcohol consumption and BMI were associated with blood loss in HTO, *R*^2^ = 0.451, *F*(9, 38) = 3.462 (*P* < 0.05). Our study indicates that alcohol consumption and BMI are important risk factors related to blood loss in HTO.

## 1. Introduction

High tibial osteotomy (HTO) is an effective procedure for knee osteoarthritis with varus malalignment in young people [1, 2]. As joint preservation surgery, the procedure moderates the mechanical axis of the lower limb to relieve pain [3–8].

HTO is usually performed with tourniquet intraoperative in order to keep a clear vision [9–12]. However, abnormal fibrinolysis induced by tourniquet release increased perioperative blood loss [13–15]. Severe hemorrhage may lead to hematoma, stiffness, infection, delayed healing, and thrombosis [9, 16–20].

Gunter et al. studied 55 patients with HTO and pointed out that the incidence of hematoma was 4.7%, infection was 4.7%, and thrombosis was 2.3% [20]. Seo et al. studied 167 patients undergoing HTO with the TomoFix plate and reported that the incidence of hematoma was 2.4% and stiffness was 1.2 [18]. Martin et al. studied 323 patients undergoing HTO with Puddu or TomoFix plate and reported that the incidence of hematoma was 3%, delayed healing was 12%, cellulitis was 10%, stiffness was 1%, and thrombosis was 1% [19]. Therefore, accurate preoperative prediction of blood loss in high tibial osteotomy is a matter of great concern to surgeons.

This study retrospectively analyzed 48 patients undergoing HTO from August 2018 to August 2019. The risk factors were studied to provide a basis for assessing blood loss before surgery.

## 2. Methods

### 2.1. Patient Selection

We retrospectively reviewed patients undergoing HTO from August 2018 to August 2019. The inclusion criteria were (1) symptomatic medial osteoarthritis, (2) the first operation, and (3) clinical data is integrity. Exclusion criteria were (1) revision surgery, (2) medical disputes, (3) preoperative hemoglobin (Hb) < 90 g/L, and (4) history of thromboembolism. 48 patients were included finally. All operations were performed by the same surgeons.

### 2.2. Data Collection

The data of 48 patients were collected, including gender, age, height, weight, BMI, smoking, alcohol consumption, hypertension, diabetes, history of aspirin, and pre-postoperative Hct. The total blood volume was calculated using height and weight by the formula of Nadler et al. [21]. The blood loss was calculated by the Gross formula [22]. Collinearity statistics was used to assess multicollinearity between independent variables, and multiple linear regression analysis was used to analyze the risk factors related to blood loss in HTO. The SPSS 22.0 software was used for statistical analysis, and *P* < 0.05 was considered statistically significant.

## 3. Results

The average age of the patients was 56.6 ± 10.2 years, including 22 males and 26 females. The mean BMI was 28.5 ± 4.2 kg/m^2^, and the mean blood loss volume was 383.3 ± 181.3 mL. There were 28 patients with left knee surgery (58.3%), 13 patients with smoking (27.1%), 15 patients with alcohol consumption (31.3%), 23 patients with hypertension (47.9%), 10 patients with diabetes mellitus (20.8%), and 12 patients with history of aspirin (25.0%). The characteristics of the patients are shown in Table 1.

The multiple linear regression analysis showed that there is no autocorrelation detected in residuals (the Durbin-Watson value was 2.348), without the presence of multicollinearity (the tolerance value > 0.1 in all independent variables), the regression of standardized residual of blood loss volume follows a normal distribution closely, and the adjusted *R*^2^ value was 0.451.

The multiple linear regression model was statistically significant *F*(9, 38) = 3.462 (*P* < 0.05), BMI and alcohol consumption are risk factors related to blood loss in HTO (*P* < 0.05), the ß value of BMI was 16.0, and the ß value of alcohol consumption was 154.6 (Table 2).

## 4. Discussion

HTO is an effective procedure for knee osteoarthritis with varus deformity [23–25]. Akizuki et al. studied 94 cases undergoing lateral closed high tibial osteotomy (CW-HTO) and pointed out that the HSS score increased significantly from 60.7 ± 11.2 preoperative to 90 ± 6.9 postoperative [6]. Niemeyer et al. reviewed 69 patients undergoing medial open wedge tibial osteotomy (OW-HTO) and pointed out that the IKDC score increased significantly from 47.25 ± 18.71 preoperative to 72.72 ± 17.15 postoperative [26]. In recent years, OW-HTO has become more popular than CW-HTO with higher correction accuracy and less soft tissue intervention [5, 27–31].

Application of tourniquet, osteotomy at the metaphyseal level, and soft tissue release intraoperative may cause intra- and postoperative bleeding. Kim et al. studied 150 patients undergoing OW-HTO and pointed out that the blood loss volume was 502.4 ± 294.9 mL in the group with tranexamic acid and 882.7 ± 482.0 mL in the group without tranexamic acid [32]. Jeya et al. studied 156 patients undergoing OW-HTO and pointed out that the blood loss volume was 372 ± 36 mL in the group with tranexamic acid and 635 ± 53 mL in the group without tranexamic acid [33]. Our study showed that the blood loss volume was 383.3 ± 181.3 mL without the application of tranexamic acid in all patients.

Severe hemorrhage may lead to hematoma, infection, delayed healing, and transfusion [9, 18, 19, 32–35]. Floerkemeier et al. studied 533 patients undergoing OW-HTO and pointed out that the incidence of hematoma was 2.1%, infection was 2.1%, infected hematoma was 0.6%, and impaired wound healing was 0.4% [34]. Seo et al. studied 167 patients undergoing OW-HTO and pointed out that the incidence of hematoma was 2.4%, deep infection was 0.6%, and nonunion was 0.6% [18]. Theoretically, accurate preoperative prediction and reducing blood loss in HTO can result in less hematoma and better wound healing.

To our knowledge, this is the first study investigating the patient characteristics related to blood loss in HTO. In our multiple linear analysis, we found that BMI and alcohol consumption are risk factors related to blood loss in HTO.

BMI was associated with blood loss which has been demonstrated in other orthopedic surgeries [36–39].

Jibodh et al. pointed out that operative time is longer and blood loss is higher in patients with BMI ≥ 40 kg/m^2^ in hip arthroplasty [36]. Frisch et al. studied 2399 patients undergoing total hip arthroplasty (THA) or total knee arthroplasty (TKA) and pointed out that the estimated blood loss of the normal group was 268.2 ± 313.9 mL, the overweight group was 282.0 ± 208.7 mL, and the obese group was 330.5 ± 302.4 mL in THA (*P* = 0.001) and the estimated blood loss of the normal group was 85.7 ± 153.8 mL, the overweight group was 90.5 ± 164.6 mL, and the obese group was 89.4 ± 72.4 mL in TKA (*P* = 0.001) [39]. This could explain that the obese patients own greater overall blood volume physiologically and need larger exposure, need greater soft tissue dissection, and has the possibility of bleeding [39, 40].

Excessive alcohol consumption has been associated with adverse outcomes in orthopedic surgeries [41–43]. However, there is rare research on the relationship between alcohol consumption and blood loss. We consider that alcohol consumption leads to excessive blood loss at multiple levels, for example, abnormally low platelet numbers in the blood, impaired platelet function, and impairment of fibrinolysis. Alcohol consumption can exert direct toxic effect on platelet production, survival, and function; previous studies have shown that alcohol has negative effects on platelet-platelet interaction and can produce thrombocytopenia which seems to be the commonly cause of platelet depletion [44–46].

Our study analyzes patient characteristics related to blood loss in HTO, wishing to establish application model in order to predict the risk of blood loss preoperative and guide blood management perioperative by patient characteristics.

This study has limitations: (1) It is a small retrospectively study. (2) The research is a single-center study in which all operations were performed by the same surgeons. The results may be biased and further larger, multicenter research was needed to confirm our findings.

## 5. Conclusion

Our study indicates that alcohol consumption and BMI are important risk factors related to blood loss in HTO; the surgeons can predict the risk of blood loss by evaluating patient characteristics.

## Figures and Tables

**Table 1:** Patient characteristics.

Gender, male:female	22/26
Mean age, years	56.6 ± 10.2
Knee involving, left/right	28/20
BMI, kg/m^2^	28.5 ± 4.2
Smoking	13 (27.1%)
Alcohol consumption	15 (31.3%)
Hypertension	23 (47.9%)
Diabetes mellitus	10 (20.8%)
History of aspirin	12 (25.0%)
Blood loss volume, mL	383.3 ± 181.3

**Table 2:** Stepwise multiple linear regression models.

	ß	*P* value
Constant	81.527	
BMI, kg/m^2^	15.996	0.013
Alcohol consumption	154.625	0.010

## Data Availability

The datasets generated and/or analyzed during the current study are available from the corresponding author upon reasonable request.
